# Mass Spectrometry Detects Sphingolipid Metabolites for Discovery of New Strategy for Cancer Therapy from the Aspect of Programmed Cell Death

**DOI:** 10.3390/metabo13070867

**Published:** 2023-07-20

**Authors:** Ming Shi, Chao Tang, Jia-xing Wu, Bao-wei Ji, Bao-ming Gong, Xiao-hui Wu, Xue Wang

**Affiliations:** 1 State Key Laboratory of Genetic Engineering and National Center for International Research of Development and Disease, Collaborative Innovation Center of Genetics and Development, Institute of Developmental Biology and Molecular Medicine, School of Life Sciences, Fudan University, Shanghai 200438, China; ming1899@gdmu.edu.cn (M.S.); 22110700022@m.fudan.edu.cn (B.-m.G.); 2 Dongguan Key Laboratory of Medical Bioactive Molecular Developmental and Translational Research, Guangdong Provincial Key Laboratory of Medical Molecular Diagnostics, Guangdong Medical University, Dongguan 523808, China; 3 National Clinical Research Center for Child Health, The Children’s Hospital, Zhejiang University School of Medicine, Hangzhou 310003, China; chtang@zju.edu.cn; 4 SINO-SWISS Institute of Advanced Technology, School of Microelectronics, Shanghai University, Shanghai 200444, China; jason-wu@sjtu.edu.cn; 5 Department of Nephrology, Children’s Hospital of Fudan University, Shanghai 200032, China; 20211240007@fudan.edu.cn

**Keywords:** sphingolipids metabolism, mass spectrometry, programmed cell death, novel anti-cancer therapy, small-molecule compounds

## Abstract

Sphingolipids, a type of bioactive lipid, play crucial roles within cells, serving as integral components of membranes and exhibiting strong signaling properties that have potential therapeutic implications in anti-cancer treatments. However, due to the diverse group of lipids and intricate mechanisms, sphingolipids still face challenges in enhancing the efficacy of different therapy approaches. In recent decades, mass spectrometry has made significant advancements in uncovering sphingolipid biomarkers and elucidating their impact on cancer development, progression, and resistance. Primary sphingolipids, such as ceramide and sphingosine-1-phosphate, exhibit contrasting roles in regulating cancer cell death and survival. The evasion of cell death is a characteristic hallmark of cancer cells, leading to treatment failure and a poor prognosis. The escape initiates with long-established apoptosis and extends to other programmed cell death (PCD) forms when patients experience chemotherapy, radiotherapy, and/or immunotherapy. Gradually, supportive evidence has uncovered the fundamental molecular mechanisms underlying various forms of PCD leading to the development of innovative molecular, genetic, and pharmacological tools that specifically target sphingolipid signaling nodes. In this study, we provide a comprehensive overview of the sphingolipid biomarkers revealed through mass spectrometry in recent decades, as well as an in-depth analysis of the six main forms of PCD (apoptosis, autophagy, pyroptosis, necroptosis, ferroptosis, and cuproptosis) in aspects of tumorigenesis, metastasis, and tumor response to treatments. We review the corresponding small-molecule compounds associated with these processes and their potential implications in cancer therapy.

## 1. Introduction

**Figure 1:**
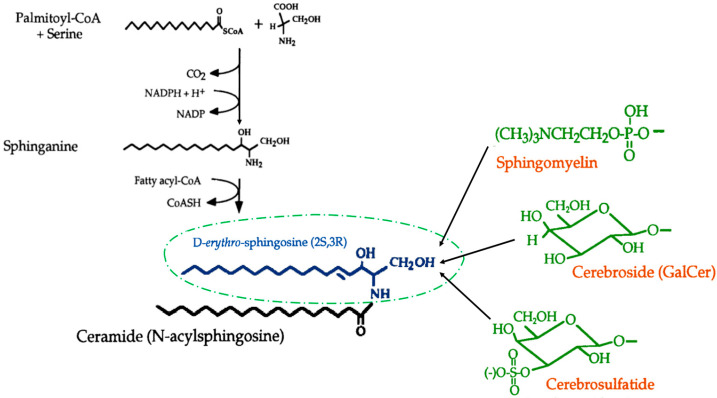
De novo biosynthesis of ceramide and primary components of sphingolipids.

Sphingolipids, present in the cell membrane of eukaryotic organisms, are a group of lipids characterized by their amino-alcohol backbones consisting of eighteen carbon atoms. The synthesis of sphingolipids primarily occurs in the endoplasmic reticulum [1]. They can also be formed from different precursors in the plasma membrane and Golgi apparatus [2,3]. In brief, the formation of ceramides, which serve as the fundamental structural units of all sphingolipids, involves the conjugation of sphingosine, derived from palmitoyl-CoA and serine, with very long-chain fatty acids. The enzymatic processes primarily catalyze sphingolipids to form sphingomyelin, cerebroside, and ganglioside (Figure 1). Sphingomyelins, which lack a glycerol backbone unlike other phospholipids, are synthesized by the transfer of phosphorylcholine from phosphatidylcholine to ceramide, resulting in the release of diacylglycerol. The cis and medial Golgi apparatus account for approximately 90% of the de novo synthesis of sphingomyelin. Although a certain amount of sphingomyelin synthesis takes place in the plasma membrane, particularly contributing to ceramide recycling, it is noteworthy that this process is the major source of sphingomyelin in oligodendrocytes and myelin membranes [4]. Cerebrosides are formed by linking either glucose or galactose to the C1 of ceramide. Mass spectrometry (MS) analysis has revealed a reduction in cerebroside levels and observed variations in fractions in numerous types of cancer [5,6]. Reliable investigation has indicated the potential significance of cerebroside variant proportions in understanding the complex biochemical and structural changes occurring during the neoplastic process. Due to structural abnormality and differentiation of various types of cells, the tumoral tissue exhibits a depleted capacity for the control of different processes concerning the synthesis of this group of lipids. Gangliosides, the most complex sphingolipids, are synthesized from galactocerebrosides and contain a huge polar head composed of several glycols, including at least one N-acetylneuraminic acid (sialic acid). The cell surface membrane is comprised of approximately 100 structurally distinct gangliosides, primarily concentrated in the brain, where they serve as crucial components [7]. Gangliosides act as differentiating markers during embryogenesis, oncogenesis and neoplastic transformation, and lymphoid differentiation. The complex sugars present on the outer surface of the membrane enable gangliosides to act as receptors for specific pituitary glycoprotein hormones that regulate essential physiological functions. Also, gangliosides can be receptors for bacterial protein toxins, such as cholera toxins. Hence, sphingolipids hold significant physiological and medicinal implications in the context of carcinogenesis. However, the metabolism of sphingolipids is subject to regulation at multiple levels and exhibits variations across different cell types. Even within a specific cell type, the regulation of sphingolipid metabolism is a dynamic process that responds to variable environmental conditions and adapts to different developmental stages. The complexity of this regulation makes it challenging to fully uncover and depict the complete story.

Progress in MS analysis has facilitated the investigation of lipid metabolism, including sphingolipids, in almost all types of tumors over recent decades. These studies have revealed that the diverse sphingolipids and enzymes catabolizing sphingolipids involved in sphingolipid catabolism possess bioactive properties, playing a crucial role in regulating cellular functions and cell death. Meanwhile, programmed cell death (PCD) mechanisms explaining how tumor cells evade death are increasingly understood. Programmed cell death, a genetically regulated process known as apoptosis, is integral to the development, homeostasis, and integrity of multicellular organisms. Programmed cell death can refer to apoptosis, autophagy, pyroptosis, necroptosis, ferroptosis, and cuproptosis [8,9]. Numerous clinical trials focusing on targeting signaling pathways involved in PCD have emerged, evaluating strategies to modulate PCD pathways in different types of tumors. This review will discuss potential therapeutic targets derived from sphingolipid biomarkers detected through MS and their incorporation into PCD signaling pathways.

## 2. Mass Spectrometry Detected Sphingolipid Biomarkers for Carcinoma

Sphingolipids, a class of endogenous biologically active molecules with diverse structures, exhibit associations with various diseases due to metabolic disorders within this lipid category [10,11,12,13,14,15,16,17]. The comparative analysis of sphingolipid metabolic networks under different physiological conditions is crucial in identifying key differential metabolites involved in regulating sphingolipid metabolism. This method is important for interpreting the underlying mechanisms of sphingolipid metabolism in various diseases and tumors [18]. The measurement of these distinct sphingolipids with different fatty acyl chain lengths (as well as distinct long-chain base sphingosine backbones) in cancer cells, tumor tissues, or plasma is performed by quantitative sphingolipidomics using mass spectrometry-based assays [19]. Mass spectrometry (MS) offers enhanced sensitivity and enables the comprehensive analysis of a wide range of cell sphingolipid metabolites, making it an invaluable tool for studying sphingolipid metabolism [20]. Researchers currently have three strategies for studying sphingolipids: untargeted, targeted, and quasi-targeted metabolomics [21,22,23]. Untargeted metabolomics refers to conducting a comprehensive unbiased analysis of all measurable sphingolipids present in the sample without understanding the composition of the sample. It aims to extract new molecular information about sphingolipids by mining extensive data, maximizing the overall understanding of the sample’s sphingolipid metabolic characteristics [24]. Targeted metabolomics mainly aims to verify whether specific sphingolipids exist in the sample and involves a selective analysis of the target sphingolipid molecules. This approach is highly focused, facilitating easier operation and analysis [25]. The combined use of these methods significantly improves information coverage and quantitative accuracy [26,27]. Below, we have summarized the specific methods and usage of MS in current sphingolipids research (Table 1).

### 2.1. Electron Ionization Mass Spectrometry 

An electron ionization (EI) source in mass spectrometry employs electrons of specific energy to directly interact with sample molecules, resulting in their efficient ionization [28]. The advantages of EI include its ease of implementation, the reproducibility of mass spectra, and the provision of more fragment ion information in the obtained results. More importantly, EI can distinguish the isomers of sphingolipids [29,30], thereby facilitating speculations regarding the structure of unknown substances. In early studies of sphingolipids, the structures of ceramide and glucosylceramide were mainly determined through EI sources [31]. However, the excessive amount of cleaved fragment ions produced by this method posed challenges in effectively analyzing the constituents of complex mixtures of sphingolipids, necessitating prior chromatographic separation to enhance analytical capabilities [32].

### 2.2. Secondary Ion Mass Spectrometry 

Secondary mass spectrometry is a soft ionization method that involves bombarding the surface with high-energy primary particles, followed by the analysis of secondary ions generated for mass spectrometry analysis [33,34]. This method is divided into fast atom bombardment mass spectrometry (FAB-MS) and liquid secondary ion mass spectrometry (LSI-MS). FAB-MS utilizes atomic beams as high-energy primary particles, whereas LSI-MS employs ion beams for the same purpose [35]. The generated excimer ions can be directly used to analyze complex sphingolipids such as ceramide, hexosylceramides, and sphingomyelin through the direct ionization of sphingolipids via high-energy atom or ion collisions. This approach yields crucial data for the structural analysis of sphingolipids [36]. This represents a significant advancement in sphingolipid metabolism research facilitated by mass spectrometry techniques.

### 2.3. Electrospray Ionization Mass Spectrometry 

Electrospray ionization (ESI) uses a strong electrostatic field to produce highly charged droplets after repeated solvent volatilization and droplet splitting. A single multi-charged ion is formed through multiple cycles of solvent volatilization and droplet fragmentation. During the ionization progress, multiple protonated ions are generated [37]. Due to the possibility that sphingolipid molecules can be directly ionized in ESI mass spectrometry [38], ESI-MS can detect and analyze a wide variety of sphingolipid metabolites, including ceramide, sphingomyelin, cerebroside, phosphatidylserine, phosphatidylinositol, and dozens of others [39].

### 2.4. Liquid Chromatography and Electrospray Ionization Mass Spectrometry 

The combination of liquid chromatography (LC) and mass spectrometry yields many advantages. LC efficiently purifies sphingolipid metabolites, isolating them from complex mixtures while reducing isomer interference and thereby improving the accuracy and sensitivity of subsequent mass spectrometry analysis. By utilizing this combination, one can accurately identify various sphingolipids such as ceramide, hexosylceramides, lactosylceramide, sphingomyelin, sphingolipid phosphorylation products, and other complex sphingolipids [40,41,42,43,44]. LC can adopt two separation modes: normal-phase chromatography and reverse-phase chromatography. Normal-phase chromatography separates compounds, such as sphingomyelin and glycosphingolipids, based on the polarity of their heads. On the other hand, reverse-phase chromatography separates these compounds by considering the carbon content and unsaturation of the main chain of sphingosine and the side chains of fatty acids [45,46,47].

### 2.5. Gas Chromatography Mass Spectrometry 

Gas chromatography (GC) is a highly efficient chromatographic technique extensively employed for analyzing volatile lipid compounds. It serves as a prevalent method for analyzing fatty acids and cholesterol [48,49]. It is necessary to perform silanization or derivatization through esterification to analyze non-volatile compounds using GC. However, this requirement restricts the application of GC in lipidomics. The GC-MS method is a widely employed technique for detecting the composition of long-chain bases and fatty acids in sphingolipid samples and conducting structural analysis. While the GC-MS method is known for its accuracy and user-friendliness, one drawback is the requirement for sample pre-treatment, which can compromise structural integrity [49].

### 2.6. Fourier Transform Ion Cyclotron Resonance Mass Spectrometry 

Fourier transform ion cyclotron resonance mass spectrometry (FTICR-MS) is a high-resolution mass spectrometry technique known for its exceptional mass resolution and accuracy. It enables the identification of small mass errors in ion peaks [50]. Therefore, Fourier transform ion cyclotron resonance mass spectrometry can effectively remove the interference of isomers and isotope lipids in sphingolipids analysis. Consequently, it enables a more comprehensive component analysis of sphingolipid metabolites, facilitating the identification and structural determination of complex glycosyl sphingolipids [51], Additionally, FTICR-MS allows for the differentiation of various types of phosphate esters [52].

### 2.7. Matrix-Assisted Laser Desorption Ionization Mass Spectrometry

In matrix-assisted laser desorption ionization (MALDI), the sample is introduced into the ion source, where the matrix generates numerous molecules upon exposure to a UV laser beam. Subsequently, the analyte undergoes ionization [11]. MALDI-MS boasts fast analysis, ease of operation, and the advantage of requiring small sample sizes. The technique has proven successful in analyzing several sphingolipids, including ceramide, hex ceramide, and sphingomyelin [11,53]. MALDI-MS offers both spatial resolution and molecular specificity, making it a powerful technique for label-free tracking of endogenous and exogenous compounds. It is particularly useful for visualizing the spatial location of lipids in biological tissues [54,55].

With the development of mass spectrometry technology, it has provided a powerful tool for analyzing and identifying aspects of sphingolipid metabolism. Currently, mass spectrometry technology enables the identification of a wide range of essential compounds within sphingolipid metabolism networks and cell signal transduction. These compounds include the fatty acids-coenzyme, ceramide, sphingosine, sphingomyelin, galactosylceramide, among others [56]. Due to the occurrence of identical ion pairs or isotope peaks in mass spectrometry analysis among certain sphingolipid compound skeletons, it is essential to perform liquid-phase separation on the sample. This step is crucial in reducing false positive interference and enhancing detection sensitivity [57,58].

## 3. Sphingolipid Metabolism and Programmed Cell Death

Programmed cell death, an active and orderly form of cell death, is ubiquitous in the cell biological process and plays a crucial role in the process of tumor cell escape. Specifically, PCD refers to the suicidal protective measures initiated by gene programming and molecular mechanisms when organisms encounter internal and external stimuli in which non-essential cells undergo specialization and removal. Malignant tumor cells must surmount diverse types of cell death mechanisms to facilitate metastasis and recurrence. Relying on interactions with membrane proteins in the plasma membrane, sphingolipids form membrane microdomains with regulatory functions, thereby engaging in intricate interactions with nearly all PCD pathways (Table 2). There is no doubt that the association of sphingolipids and PCD pathways will provide new insights into cancer treatment. Here, we mainly discuss the relevance between sphingolipids and cell apoptosis, autophagy, pyroptosis, necroptosis, and ferroptosis, as well as the newly discovered cuproptosis.

### 3.1. Apoptosis

Specific cell death signaling pathways activate apoptosis. These pathways involve cytoplasmic shrinkage, budding of the plasma membrane, eversion of phosphatidylserine (PS), chromatin condensation, and DNA breakage [59,60], which gives rise to cell clearance [61]. During the whole process of apoptosis, the cell membrane remains intact, preventing the release of cellular endocytes and the onset of inflammatory responses [62]. There are two main pathways of apoptosis: the extrinsic receptor-mediated pathway and the intrinsic mitochondrial pathway. Extrinsic receptor-mediated pathways refer to death ligands such as tumor necrosis factor (TNF), Fas, and members of the tumor necrosis factor receptor family and their related ligands [63]. These ligands trigger the formation of a death-induced signaling complex (DISC) that recruits multiple effectors to facilitate cellular apoptosis. Sphingolipids play a crucial role as a regulator in the extrinsic receptor-mediated pathway of apoptosis. Sphingomyelinase hydrolyzes raft sphingomyelin to ceramide, and ceramide molecules spontaneously associate to form microdomains with the enrichment of ceramide. These ceramide-enriched microdomains can reorganize receptors and signaling molecules present both inside the cells and on the cell membranes, thereby facilitating and amplifying specific receptor-mediated signaling transduction [64]. An example of such modulation is observed in the acid sphingomyelinase (aSMase)-mediated aggregation of CD95 within ceramide-rich membrane platforms. This aggregation event is crucial for DISC formation [65,66,67]. In addition, the binding of ligands with receptors, such as the p55 TNF receptor, interleukin-1 receptor, and Fas receptor, activates SMase, resulting in the generation of ceramide. Ceramide then functions as a second messenger, exerting regulatory control over apoptotic signaling pathways [68,69,70,71]. Multiple signaling pathways are transduced and aggregated to mitochondria, leading to the induction of mitochondrial outer membrane permeability (MOMP). This phenomenon is recognized as intrinsic mitochondrial-mediated apoptosis. The release of intercellular space proteins, including cytochrome c and Apaf1, triggers the activation of caspases and DNases within the cytoplasmic matrix. These proteins play a crucial role in regulating cell death processes [72]. The anti-apoptotic and pro-apoptotic members of the Bcl-2 family are highly homologous in one or more specific domains and regulate the process of MOMP. The overexpression of anti-apoptotic Bcl-2 members protects against apoptosis induced by various stimulus signals [73]. Various key enzymes in regulating sphingolipid metabolism exist in mitochondria, such as ceramide synthase, ceramidase, sphingosine kinase, and neuraminidase 4 [74,75,76]. Studies have provided evidence of elevated ceramide levels preceding the mitochondrial phase of apoptosis [77,78,79]. Moreover, increased ceramide levels are observed in response to various apoptotic stimuli [80,81]. Interestingly, as the metabolic content of ceramide decreases, cells can also avoid apoptosis. For example, glucosylceramide synthase (GCS) converts ceramide metabolism into glucosylceramide. Downregulating ceramide levels through this process provides cellular protection against ceramide-induced apoptosis and enables the cells to develop resistance against apoptosis, promoting anti-apoptotic responses [82]. As another ceramide metabolite, sphingosine-1-phosphate (S1P) and its metabolites exert the opposite effect on apoptosis: on the one hand, activating extracellular regulated protein kinases (ERK) and other anti-apoptotic signaling pathways inhibits apoptosis induced by ceramide [83,84,85]; on the other hand, S1P and its metabolite, acetaldehyde, can also promote apoptosis by activating the pro-apoptotic Bcl-2 proteins Bak and Bax [86,87].

**Figure 2:**
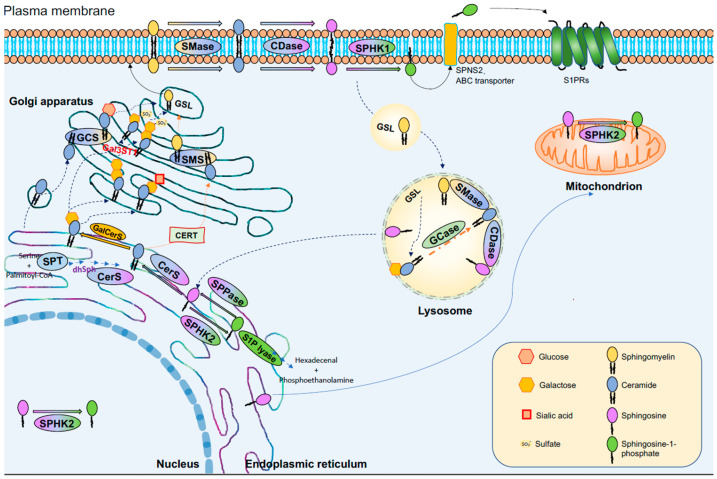
Subcellular compartmentalization of sphingolipid metabolism.

Ceramide protects cells from apoptosis in certain cancer cells [88]. C16 ceramide protects head and neck squamous cell carcinoma (HNSCC) cells from the endoplasmic reticulum (ER) stress-mediated apoptosis [89]. Conversely, C24 ceramide protects HeLa cells against apoptosis induced by ionizing radiation (IR). However, in the case of cancer cells, C16 ceramide promotes apoptosis mediated by IR [90]. One possible explanation could be that the regulation of apoptosis by ceramide is affected by both the subcellular localization of ceramide and the type of stress stimulation (Figure 2). These factors contribute to the contrasting outcomes, either promoting or inhibiting apoptosis.

### 3.2. Autophagy

Autophagy is a highly conserved cellular degradation process in eukaryotes, responding to various stimuli to maintain the balance of cells, tissues, and organisms [91]. Autophagy can be divided into three types: macro-autophagy, micro-autophagy, and chaperone-mediated autophagy (CMA) [91,92,93]. The whole process of autophagy includes the initiation of autophagosomes, the nucleation of membranes, the expansion and extension of the autophagosome membrane, the closure of the autophagosome membrane, its fusion with lysosomes, and ultimately, the degradation of the autophagic contents [94,95]. When the body is under appropriate stimulation, the phosphorylation of the core complex ULK1 in the autophagy process is prevented. This cessation of ULK1-complex inhibition triggers the initiation of autophagosome formation [96,97]. The ULK1 complex activates another complex, Beclin-1, and enhances the activity of VPS34, which is located in PI3K. This activation leads to the formation of autophagosome membrane PI3P and the recruitment of crucial factors for autophagy induction, such as double FYVE-containing protein 1 (DFCP1) [98,99]. When the autophagosome matures, it undergoes fusion with the outer membranes of lysosomes, resulting in the degradation of the cargo transported by lysosomes. The involvement of sphingolipids in autophagy is evident across various stages of the autophagic process. In the early stage of autophagy, Beclin-1, an important complex, binds to Bcl-2, resulting in the inhibition of Beclin-1 [100], while ceramide can induce the disassociation of Beclin-1 from the complex and the release of Beclin-1. This release of Beclin-1 plays a vital role in initiating autophagy [101]. In addition, the change in sphingolipid levels in the plasma membrane regulates the levels and activity of nutrient transporters [102,103]. These transporters play a critical role in controlling the cellular fuel supply. Conversely, ceramides downregulate the expression of amino acids and nutrient transporters, leading to cellular starvation. This, in turn, triggers the induction of survival autophagy through the reduction in mTOR signaling transduction or activation of AMPK signaling [104,105]. During autophagy, autophagosomes engulf various cytoplasmic components, as well as mitochondria, the endoplasmic reticulum, and peroxisomes [106]. In mitochondrial autophagy, ceramides can diminish the mitochondrial membrane potential, resulting in mitochondrial dysfunction. Furthermore, this process promotes the expression of the mitochondrial-associated cell death protein BNIP3 [107]. The ectopic expression of Cers1 leads to the formation of autophagosomes that directly bind to ceramides on the mitochondrial membrane [108]. Autophagosomes and lysosomes associate to form autolysosomes that degrade the substance regulated by sphingolipids [109]. More recently, it has been found that ceramide-1-phosphate, derived from sphingomyelin by sphingomyelinase and ceramide kinase, promotes calcium-dependent liposome fusion and regulates the fusion of the autophagosome and lysosome [110].

### 3.3. Necroptosis

Necrotic apoptosis, also known as necrotic apoptosis, represents an alternative form of programmed cell death. During necroptosis, apoptotic cells undergo rapid swelling, accompanied by concurrent organelle swelling [111]. Necroptosis involves key players such as RIPK3 and its substrate MLKL, whose interaction with lipids is a key step in the implementation of necroptosis [112,113]. Ceramide can target MLKL to activate necroptosis independent of the RIPK1/RIPK3 regulatory pathway in ovarian cancer [114]. Furthermore, during necroptosis, MLKL translocates to lipid rafts in the plasma membrane, and ceramide facilitates the relocation of MLKL to these lipid rafts [115]. Ceramide can form a complex with receptor-interacting Ser/Thr kinase 1 (RIPK1) and form a ceramide-enriched membrane pore channel mediated by non-muscle myosin IIA. In this process, ceramide binds to specific residues, such as ASP147 and ASN169 of RIPK1, while the interaction with myosin IIA involves the residues Arg258 and Leu293 [116].

### 3.4. Pyroptosis

Pyrosis is an inflammatory cell death in that various pathological factors, including related signals can trigger and activates Caspase-1 through classical inflammatory corpuscle signaling pathways or activates Caspase-4, -5, -11 through non-classical pathways to exert various downstream effects [117]. Ceramide is mainly located in the endoplasmic reticulum membrane and produces sphingosine through ceramidase. On the one hand, sphingosine translocates from the endoplasmic reticulum (ER) membrane to the cell membrane, where it undergoes conversion into sphingosine-1-phosphate (S1P) mediated by sphingosine kinase 1 (SPHK1). The buildup of sphingosine within the ER can trigger the activation of NLRP3 and oligomeric RLRP3 inflammasomes, leading to the homogeneous activation of Caspase-1 and subsequent production of IL-1/IL-18 [118]. In addition, when lysosomes are destroyed, Cathepsin B diffuses into the cytoplasmic matrix and cleaves SPHK1 [119]. Therefore, sphingosine-induced lysosomal membrane rupture leads to Cathepsin B translocation from the lysosome to the cytoplasm, which results in the cleaved SPHK1 and the inhibition of sphingosine conversion to S1P. As a result, the accumulated sphingosine activates the assembly of NLRP3 inflammasomes, ultimately inducing pyroptosis [118].

### 3.5. Ferroptosis and Cuproptosis

Ferroptosis is a form of cell death that relies on ferric ions to generate reactive oxygen species (ROS). These ROS initiate alterations in metabolic processes and the accumulation of lipid peroxides, leading to structural damage to cell membranes and ultimately resulting in cell death [120]. GXP4 is a defense inhibitor against LPO and iron apoptosis [121]. In cases of GSH deficiency, the activity of glutathione peroxidase 4 (GPX4) decreases, leading to an increase in intracellular lipid peroxide (LPO) levels. This imbalance in cellular redox status inclines cells towards undergoing ferroptosis [122]. Ceramide accumulation is observed in the early stages of iron-induced apoptosis, coinciding with glutathione depletion and ROS production. Ceramide can be activated by various stressors, such as genotoxic damage, inflammatory mediators, heat shock, oxidative stress, and anti-cancer drugs, forming membrane platforms enriched with ceramide [123]. aSMase-mediated ceramide has been proven to induce ROS production in cells. Additionally, ceramide is known to form membrane platforms enriched with ceramide. Concurrently, NADPH oxidase aggregates and becomes activated within these membrane platforms, producing ROS. As the ROS levels increase, the Cys-629 residue located in the aSMase undergoes oxidation. This oxidation event promotes the dimerization and activation of aSMase, facilitating the generation of more ceramide. This ceramide undergoes repeated circulation within the system [124,125]. Furthermore, aSMase also facilitates GPX4-dependent autophagy, an essential process associated with ferroptosis. Previous studies have shown that ferroptosis is accompanied by autophagy activation, while aSMase-mediated autophagy plays a key role in ferroptosis [124,126,127].

Cuproptosis is a recently identified form of cell death specifically triggered by copper. The mechanism underlying cuproptosis distinguishes it from all other known cell death pathways. Cuproptosis occurs due to the interaction between copper and the fatty acylation components within the tricarboxylic acid (TCA) cycle. This association leads to the buildup of fatty acylated proteins and the depletion of iron–sulfur cluster proteins. Consequently, protein toxic stress ensues, ultimately culminating in cell death [128]. At present, limited research exists on cuproptosis, and the relationship between sphingolipid metabolism and cuproptosis remains largely unknown. Further investigations are needed to elucidate this relationship. Experimental evidence has confirmed that copper can induce apoptosis by aSMase and subsequently trigger the release of ceramide [129,130]. Cu^2+^ initiates hepatocyte apoptosis by the activation of aSMase and subsequent release of ceramide. Additionally, Cu^2+^ induces the secretion of activated aSMase in leukocytes, leading to ceramide release and phosphatidylserine exposure in erythrocytes. This exposure facilitates the rapid clearance of affected erythrocytes within the bloodstream of mice [131].

**Table 1:** Sphingolipids detected by MS methods.

Mass Spectrometry	Advantages, Disadvantages, and Characteristics	Sphingolipids	Ref.
LC-MS, HPLC-ESI-MS	Detect and analyze a wide variety of sphingolipids metabolites	Ceramide	[40,56,132,133]
FTICR-MS, HPLC-ESI-MS	Detect and analyze a wide variety of sphingolipids metabolites	Sphingosine	[40,56]
FTICR-MS, LC-MS	Remove the interference of isomers and isotope lipids in sphingolipid analysis	Sphingomyelin	[56]
FTICR-MS with LC-MS	Reduce false positive interference and improve detection sensitivity	Dihydroceramide	[134,135]
LC-MS	Reduce the interference of isomers, improve the accuracy and sensitivity	Glucosylceramide	[136,137]
LC-MS/MS	Reduce the interference of isomers, improve the accuracy and sensitivity	Sphingosine-1-phosphate	[138]
LC-MS	Reduce the interference of isomers, improve the accuracy and sensitivity	Phosphatidylinositol	[139]

**Table 2:** Sphingolipids interplay with PCD pathways.

Cell Death Pathway	Sphingolipid	Morphological Feature	Function	Ref.
Apoptosis	Ceramide, sphingomyelinase phosphate	Induces the extrinsic pathways of the apoptosis pathway	Adjusted, amplified the signal	[64,65,66,67]
	Ceramide	Induces the mitochondrial intrinsic apoptosis pathway	Induced	[140]
	Dihydroceramide	Interferes with the formation of ceramide channels in mitochondria, significantly reduces the permeability of the outer mitochondrial membrane, and inhibits ceramide-induced apoptosis	Inhibited	[141]
	Ceramide synthase 6 (CerS6), C16-Ceramide	Regulates activation of ER stress response	Inhibited	[89]
	Glucocerebroside; glycosphingolipid, (GSL); Glucosylceramide Synthase (GCS)	Induces mitochondrial intrinsic apoptosis pathway	Anti- Apoptosis	[82]
	S1P	Activates ERK and other signaling pathways	Anti- Apoptosis	[83,84,85]
	S1P	Activates pro-apoptotic Bcl-2 proteins Bak and Bax	Pro- apoptosis	[86]
Autophagy	Ceramide	Downregulates nutrient transporters	Promoted	[102,104,105]
		Induces the release of Beclin-1 by dissociation of the Beclin-1/Bcl-2 complex	Induced, promoted	[101]
		Mediates autophagosomes directly anchor to mitochondria	Promoted	[108]
		Reduces mitochondrial membrane potential and activates BNIP3 transcription	Induced	[107]
	Dihydroceramide desaturase 1, DES1	ATP synthesis damage activates Ampk, activating unc-51-like kinases to cause autophagosome formation	Promoted	[142]
	Sphingomyelin, sphingomyelinase, ceramide kinase, ceramide-1-phosphate	Calcium-dependent liposome fusion, which regulates the fusion of autophagosomes and lysosomes	Promoted	[110]
	Sphingosine-1-phosphate phosphohydrolase-1 (SPP1)	Deletion of SPP1 increases the expression of transcriptional regulators C/EBP homologous protein and Grp78/BiP, as well as phosphorylation of eukaryotic translation initiation factor-2 α (eIF2α), induces ER stress.	Induced	[143]
Necroptosis	Ceramide	Mediates MLKL repositioning into lipid rafts	Induced	[115]
		Ceramide–RIPK1 complex formation leads to disruption of lipid bilayer integrity	Induced	[116]
Pyroptosis	Sphingosine, SPHK1	Activates NLRP3 and oligomeric RLRP3 inflammasomes	Induced	[118]
Ferroptosis	Ceramide; acid sphingomyelinase, aSMase	Ceramide enrichment membrane plateau formation induces GPX4 autophagy degradation	Induced, promoted	[124]

**Table 3:** Inhibitors focusing on sphingolipid-motivated PCD in preclinical or clinical phase.

Inhibitor/Drug Name	Target Sphingolipids	Related PCD	Cancer Relevance	Trial Phase	Ref.
Myriocin	De novo ceramide synthesis	Apoptosis	Increased activity in response to chemotherapy and radiotherapy in breast cancer cells		[144,145,146]
FB1; HDAC1 or HDAC2	Synthesis of C18 (dihydro)ceramide	Autophagy	Induces mitophagy in head and neck and AML cell lines, mouse xenograft models, and patient-derived AML cells	Preclinical	[147,148,149]
FB1	Synthesis of C16 (dihydro)ceramide	Apoptosis—mitochondria	Induces caspase activation and cell death in lung cancer cells; preserves ER and Golgi integrity in head and neck cancer cells; elevated in breast tumour tissues; protects from GVHD in a mouse model of leukaemia	Preclinical	[89,150,151,152,153,154,155,156]
Fenretinide; ABC294640; C8-CPC	Ceramide synthesis	Apoptosis	Induces cell cycle arrest in neuroblastoma cells	Preclinical	[157]
Tri-cyclic anti-depressants	Ceramide generation	Apoptosis	Induces apoptosis in lymphoblasts; promotes haematogenous tumour metastasis in mouse models		[158,159,160]
GW4869	Ceramide generation	Apoptosis	Mediates cell cycle arrest in breast cancer cells; exosome release		[161,162,163]
THI	S1P breakdown	Apoptosis	Induces ceramide accumulation and colon cancer cell death		[164,165]
CHC	Ceramide transport from ER to Golgi	Autophagy	Inhibits pro-apoptotic ceramide signalling in breast cancer cells and tumours in mouse models	Preclinical	[166,167,168]
NVP-231	C1P generation	Apoptosis	Induces breast cancer cell survival in culture and mouse models	Preclinical	[169,170,171]
PPMP; PDMP	GlcCer synthesis	Apoptosis—mitochondria	Mediates drug resistance in patients with oral cancer and in breast cancer cells and xenografts		[172,173,174,175,176]
LCL-521	Ceramide cleavage	Apoptosis—mitochondria	Mediates resistance to cell death in prostate cancer cells and xenografts and elevated in tumours from patients	Preclinical	[177,178,179,180]
PF543	S1P generation	Apoptosis	Mediates pro-survival signalling and metastasis in bladder cancer, lung cancer, and melanoma cells in culture and in mouse models	Preclinical	[181,182,183]

## 4. Conclusions and Prospects

Advancements in genetic and molecular techniques have led to significant discoveries unveiling the involvement of sphingolipids in cancer progression and treatment over the past several decades. In particular, mass spectrometry is an analytic technology innovated and widely used to quantitate and distinguish specific sphingolipid metabolites relevant to cancer cells, cancer patients’ peripheral blood, tumor tissues, and adjacent tissues or cells near tumor cell clusters. With the deep lipidomic excavation using MS, sphingolipid metabolites have been identified as crucial players in diverse signaling pathways implicated in cancer development, evasion of stem cells, drug resistance, and even modifications in drug response. The distinctive physiological functions of sphingolipids offer a novel avenue for studying cellular pathology and disease mechanisms, presenting exciting opportunities for further research. Mechanistically, most enzymes involved in sphingolipid metabolism can generate genetic models to study their roles in cancer signaling pathways. Additionally, these models contribute to developing and designing structure–function-based anti-cancer drugs, opening new avenues for therapeutic advancements. The synthesis and accumulation of sphingolipids can mediate cancer cell death through various mechanisms, including induction of apoptosis, necroptosis, autophagy, ferroptosis, and cellular stress like ER stress [88]. Several inhibitors specifically targeting sphingolipid-driven PCD have been developed and extensively investigated (Table 3). Many cancer treatments targeting the sphingolipid metabolism pathway have achieved clinical results. Fenretinide can target DES enzymes to reduce the de novo synthesis of ceramides and regulating the increase in dihydroceramide levels. But, there are some side effects after medication, including diarrhea, allergic reactions, and others [184]. One Phase III clinical trial of fenretinide showed no significant difference between treatment groups, accompanied by certain visual problems and toxic side effects of musculoskeletal disorders [185]. In addition, fenretinide showed insufficient antitumor activity, such as against breast cancer [186] and kidney cancer [187]. ABC294640 is an orally administered inhibitor of SPHK2 and DES1 that inhibits the synthesis of S1P, which is an anti-apoptotic sphingolipid metabolite, and increases the content of intracellular ceramides [188,189]. The current ABC294640 Phase I clinical trial results for solid tumors have shown a good tolerability; ABC294640 administration can cause S1P levels to decline in the first 12 h and return to baseline within 24 h with side effects include nausea, vomiting, and fatigue [190].

In addition, the different subcellular localizations cause sphingolipids to differentially mediate cell death pathways, varying depending on the cell and tissue type. This characteristic renders sphingolipids an appealing therapeutic target for anti-tumor interventions (Figure 2). MALDI visualizes and images the tissue localization of these distinct sphingolipids. Based on these techniques, ceramide and S1P are defined by anti-cancer and pro-oncogenic functions depending on the microenvironment and cell type [191,192,193]. The intracellular bioactive sphingolipid network is extremely complex and involved in many kinds of life activities. Cytoplasmic sphingolipids located regionally can lead to functional differences; thus, in the context of the entire biological organization, the study of sphingolipids is very difficult [194]. Nevertheless, the emergence of new technologies has provided various contributions to clarify the mechanism of sphingolipids in various life activities [88]. It is currently clear that different downstream targets of ceramide and S1P produce different results and, even in the same cell, sphingolipids in different subcellular structures changing will lead to different cell fate trajectories; this is why ceramide, S1P, or other kinds sphingolipid can participate in different types of PCD at the same time. In addition, sphingolipids represent components of the plasma membrane involved in regulating biofilm fluidity, forming sphingolipid-enrichment regions to form special functional platforms [124,195], and regulating cell growth and proliferation through signaling. A mitophagy-promoted cancer cell death induced by a mitochondrial accumulation of pyridinium ceramide has been discovered [196]. The intricate regulatory mechanisms of sphingolipids within different cells or organelles under varying microenvironments give rise to diverse downstream targets. These targets ultimately influence cellular fate and outcomes. For example, SPHK2-generated S1P-protein binding regulates other S1P targets by allosterically mimicking protein phosphorylation. Notably, this interaction impacts various proteins, including PPARγ, HDAC1/2, and PHB2 [197,198,199]. This process plays a critical role in controlling drug-induced senescence and cancer-associated PCD. Revealing the roles of sphingolipids in signal transduction and cross-talk between tumors and host cells (primary stromal cells, endothelial cells, osteoclasts, or platelets) can help to develop novel therapeutic strategies to inhibit cancer growth and metastasis.

However, numerous preclinical and clinical drugs have been developed and subjected to trials, capitalizing on the aforementioned advantages (Table 3). Most trials have failed or been stopped due to our limited understanding of the intricate signal transduction processes involved in cancer. The central key of cancer biology is the type and condition of cells transformed to form tumors. Therefore, effective treatment strategies should be rooted in a deep understanding of carcinoma and the overall metabolic profiles of individual patients. The cornerstone of cancer biology lies in comprehending the characteristics and status of cells that transform to give rise to tumors. Therefore, effective treatment strategies should be rooted in a deep understanding of carcinoma and the overall metabolic profiles of individual patients. Tracing and defining the roles of sphingolipids in regulating PCD versus survival is a challenge. This challenge arises from the rapid in vivo metabolic interconversions, intricate trafficking, and multifaceted signaling roles that sphingolipids assume within cell and organelle membranes. Moreover, further studies are needed to ascertain the precise mechanisms by which sphingolipids function in immunocyte-mediated tumor killing and how they influence the effectiveness of immunotherapy in combating cancer. For example, immunotherapy with ch14.18, GM-CSF, and interleukin-2 was associated with a significantly improved outcome as compared with standard therapy in patients with high-risk neuroblastoma. Rapid advancements in MS technology have paved the way for significant breakthroughs in unraveling the molecular interactions and signaling networks underlying the immune metabolism of sphingolipids. This progress holds promising prospects for the future development of improved treatments for solid tumors.
